# Adjuvant androgen deprivation impacts late rectal toxicity after conformal radiotherapy of prostate carcinoma

**DOI:** 10.1038/sj.bjc.6600266

**Published:** 2002-06-17

**Authors:** G Sanguineti, S Agostinelli, F Foppiano, P Franzone, S Garelli, M Marcenaro, M Orsatti, V Vitale

**Affiliations:** Department of Radiation Oncology, National Institute for Cancer Research, Genoa, Italy; Department of Physics, National Institute for Cancer Research, Genoa, Italy

**Keywords:** prostate carcinoma, adjuvant androgen deprivation, conformal radiotherapy, late rectal toxicity

## Abstract

To evaluate whether androgen deprivation impacts late rectal toxicity in patients with localised prostate carcinoma treated with three-dimensional conformal radiotherapy. One hundred and eighty-two consecutive patients treated with 3DCRT between 1995 and 1999 at our Institution and with at least 12 months follow-up were analysed. three-dimensional conformal radiotherapy consisted in 70–76 Gy delivered with a conformal 3-field arrangement to the prostate±seminal vesicles. As part of treatment, 117 patients (64%) received neo-adjuvant and concomitant androgen deprivation while 88 (48.4%) patients were continued on androgen deprivation at the end of three-dimensional conformal radiotherapy as well. Late rectal toxicity was graded according to the RTOG morbidity scoring scale. Median follow up is 25.8 (range: 12–70.2 months). The 2-year actuarial likelihood of grade 2–4 rectal toxicity was 21.8±3.2%. A multivariate analysis identified the use of adjuvant androgen deprivation (*P*=0.0196) along with the dose to the posterior wall of the rectum on the central axis (*P*=0.0055) and the grade of acute rectal toxicity (*P*=0.0172) as independent predictors of grade 2–4 late rectal toxicity. The 2-year estimates of grade 2–4 late rectal toxicity for patients receiving or not adjuvant hormonal treatment were 30.3±5.2% and 14.1±3.8%, respectively. Rectal tolerance is reduced in presence of adjuvant androgen deprivation.

*British Journal of Cancer* (2002) **86**, 1843–1847. doi:10.1038/sj.bjc.6600266 www.bjcancer.com

© 2002 Cancer Research UK

## 

Several trials have shown some advantage of adding androgen deprivation (AD) to conventional radiotherapy to 66–70 Gy for prostate carcinoma (Bolla *et al*, 1997; Lawton *et al*, 2001; Zagars *et al*, 1988; Granfors *et al*, 1998) although improved overall survival was observed only in one (Bolla *et al*, 1997).

On the other hand, a prospective trial suggests that, when localised prostate cancer is treated by radiotherapy alone, dose escalation to the total dose of 78 Gy with three-dimensional conformal radiotherapy (3DCRT) leads to improved outcome over 70 Gy (Pollack *et al*, 2000). However, despite ameliorations in radiotherapy treatment planning and delivery, late toxicity on both bladder and rectum remains a limiting factor in dose escalation for prostate carcinoma due to the proximity of these organs to the target.

The next logical step would be the combination of the two modalities, radiotherapy dose escalation and adjuvant AD (AAD), as it is currently being investigated by the European Organisation for Reseach and Treatment of Cancer within two separate phase III studies (22991 and 22961). However, whether the addition of AAD to high-dose 3DCRT reduces rectal tolerance and, therefore, whether radiotherapy dose escalation is feasible in presence of AAD is essentially an unaddressed issue.

While some quoted studies did not even mention late toxicity (Pollack *et al*, 1995; Zagars *et al*, 1999; Laverdiere *et al*, 1997), some other reports may suggest a reduced tolerance of both genitourinary and gastrointestinal systems in presence of AAD (Bolla *et al*, 1997; Fiorino *et al*, 2001; Sanguineti *et al*, 2000).

In a previous analysis we had found that AAD was associated with an increased risk of late rectal toxicity (Sanguineti *et al*, 2000). However, in that analysis, due to a large time-span, an heterogeneous population of patients with localised prostate cancer was considered. Moreover, treatment related parameters, that can be influenced by AD, were not taken into account. Thus, in the present paper we focused on a homogeneously treated patients for whom technical details of treatment were prospectively recorded.

## MATERIALS AND METHODS

### Patient population

We analysed 188 consecutive patients with prostate cancer, treated at our institution with 3DCRT on the prostate±seminal vesicles from 1995 to 1999. Of all patients referred to us during this time period for radical treatment, we only excluded six patients without 12 months minimum follow-up, because of early death due to intercurrent causes (4 patients) or distant metastases (2 patients) thus leaving 182 patients for analysis. According to the 1997 UICC staging system, six (3.2%) patients were classified as T1b, 40 (22%) as T1c, 66 as T2a (36.3%), 26 (14.3%) as T2b, 30 as T3a (16.5%) and 14 (7.7%) as T3b. The median age was 71.5 years ranging from 50–83 years. No patient had evidence of pelvic lymph node involvement at diagnosis (N0–Nx).

### Treatment strategy

Our prescription dose for T⩾1b prostate cancer has changed during years. From 1995 to 1998, only patients with T⩾2a prostate carcinoma were prescribed 76 Gy, with 70 Gy to T1 stages. From 1999 all patients with primary tumour staged ⩾1b were administered 76 Gy. The only exception to higher dose RT was represented by patients with diabetes mellitus and severe cardiovascular disease, who were always prescribed 70 Gy.

Regarding target volumes, treatment was limited to the prostate±seminal vesicles (SV). For SV inclusion we followed the guidelines reported by Katcher *et al* (1997). The whole pelvis was never treated.

In our experience, neoadjuvant hormonal therapy prescription was related mainly to the referring urologist rather than the volume of prostate gland at diagnosis (Zelefsky and Harrison, 1997). Patients undergoing neoadjuvant AD were typically prescribed monthly or trimonthly LH–RH analogue preceded by 2–3 weeks of anti-androgens. In fact, we collect patients from three different urology departments and several private practices; they refer us patients after neoadjuvant AD has been already started. This along with the fact that data about the efficacy of neoadjuvant AD changed during years (Pilepich *et al*, 1995; Laverdiere *et al*, 1997), resulted in a wide spectrum of indications and duration of neoadjuvant AD. As result, all but 65 patients (117 patients, 64%) underwent neoadjuvant AD for a median duration of 4.2 months (0.4–67.2 months) before 3DCRT.

Our policy was to leave hormonal treatment during radiotherapy for those patients who had already started it. Eighty-eight (48.4%) patients with Gleason score greater than 6 or PSA at diagnosis greater than 20 ng ml^−1^ were continued on adjuvant AD for a minimum of 1 year after treatment end.

### Treatment technique

On average simulation was performed 2.1 weeks (range: 0.1–7 wks) before 3DCRT.

X-ray simulation was performed before planning CT scanning in order to define the position of the isocenter and to obtain two orthogonal (0 and 90 degrees) 10×10 cm films for reference purposes. Patients were immobilised supine in a thermoplastic device with empty rectum and bladder. The preliminary position of the isocenter was marked on the mask. Isocenter was typically positioned at mid pubic symphysis level on the midline on AP fields, and behind the femoral heads on lateral fields. A fenestration was cut in the device at the level of the transverse lasers and the patient skin was tattooed accordingly on both sides in order to better align the patient to the mask.

CT was performed with a dedicated scanner and slices were taken at 5 mm intervals covering the whole target. The clinical target volume (prostate±seminal vesicles)(CTV), the rectum (outer contour) and the bladder were drawn on CT slices. Until July 1997, only 6–10 representative CT slices were loaded in the treatment planning system (Nucletron Plato); afterwards all slices (20–25) were included.

**Figure 1:**
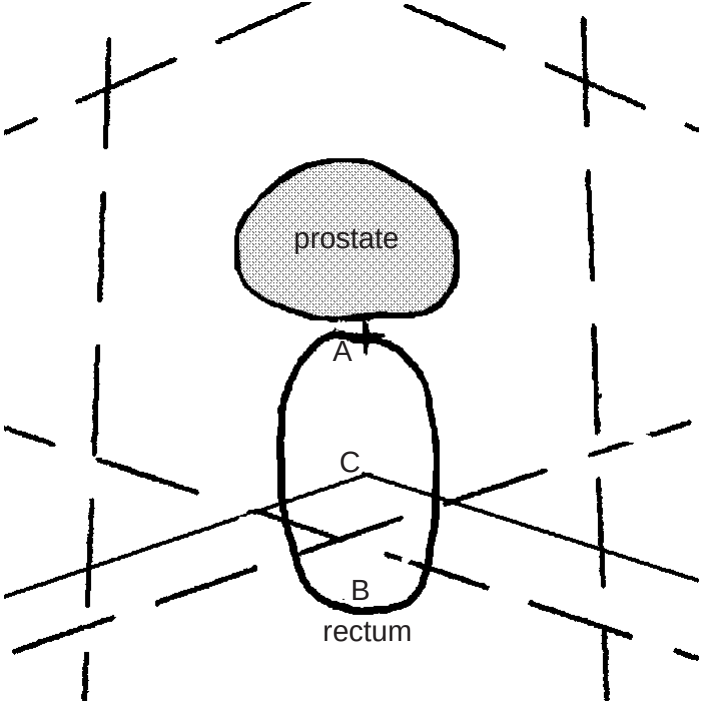
Geometrical parameters at central axis level. (**A**) anterior rectal dose; (**B**) posterior rectal dose; (**C**) depth of oblique fields; dashed lines: open field borders; solid lines: posterior edge of lateral fields as from MLC.

The planning target volume (PTV) was obtained by adding 1.3 cm to CTV except at the prostate–rectum interface where a 0.8-cm margin was used. An additional 0.5-cm margin was added circumferentially around the PTV to account for radiation beam penumbra. A 1-cm multileaf collimator was used to shape the fields according to beam's eye view findings. Our 3D conformal radiotherapy set-up involves three isocentric coplanar photon (15 MV) fields (0, 110 and 250 degrees) (Figure 1

).

The radiation dose was prescribed to the isocenter (International Commission on Radiation Units and Measurements point). When initially included in target (‘initial phase’), the seminal vesicles were excluded at 60 Gy (‘boost phase’). A dose distribution was obtained at central axis level in order to optimize beam weights. No wedges were used.

### Statistics

Patients were seen 3 months after treatment end and every 6 months afterwards. Rectal complications were scored according to Radiation Therapy Oncology Group scale (Cox *et al*, 1995). Acute ones were defined as those occurring during treatment or within 90 days from its completion. Late complications were those developing more than 90 days after treatment end or those starting prior to and persisting for longer than 90 days after completion of treatment (Table 1

**Table 1:**
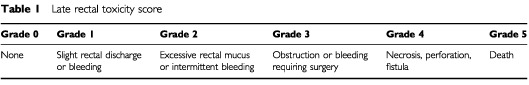
Late rectal toxicity score

).

Survival curves were calculated using the Kaplan–Meier method from the date of treatment end. Actuarial incidence of grade 2–4 reactions was analysed in relation to clinical, anatomical and dosimetric/geometric variables using univariate and multivariate analyses. Clinical variables were: age (on a continuum), diabetes mellitus (no *vs* yes), vascular comorbidity (according to Kaplan–Feinstein (Piccirillo and Feinstein, 1996)) (grades 0–1 *vs* 2–3), T stage (T1 *vs* T2 *vs* T3) and acute rectal toxicity (grades 0–1 *vs* 2–3).

On the central slice, where the isocenter was located, the following parameters were identified (Figure 1): rectal wall thickness (AB distance on a continuum); depth of oblique fields (distance between the posterior edge of oblique fields and the anterior margin of the rectum or AC distance on a continuum); depth of oblique fields normalised to rectum thickness (ratio between AC and AB on a continuum); posterior rectal wall dose (at point B on a continuum), anterior rectal wall dose (at point A on a continuum).

Other included factors were: radiation oncologist (GU dedicated *vs* occasional), neoadjuvant+concomitant AD (no *vs* yes), adjuvant AD (no *vs* yes), duration of radiotherapy (on a continuum), interval time between simulation and radiotherapy (on a continuum), target volumes (prostate *vs* prostate+SV), ICRU prescribed dose (70 Gy *vs* 76 Gy), number of slices of CT loaded in the treatment planning system (⩽10 *vs* >10).

Moreover, for both the initial and boost phases we considered the height of fields and the volume of irradiated volume (on a continuum). This was assumed to be a box whose volume is the average of the ones calculated for each field (cubic root of the product of the volume of each field). Each irradiated volume was obtained by multiplying the effective area of the corresponding field and a depth. The former was provided by computerised analysis of the multileaf file. The latter is calculated by the square root of the effective area as follows. The square root of the AP field provides an estimate of the depth of the laterals; the depth of the AP field is the average of the square root of each lateral field.

For patients treated on the prostate alone, the initial and the boost phase were considered to be the same.

Univariate analysis was performed with the log-rank test and multivariate analysis was performed using the Cox proportional hazards model with both forward and backward stepwise procedures. Variables with a *P*⩽0.2 at univariate analysis were entered in the multivariate one. Unless otherwise specified, median values have been chosen as cut-off values. In all cases, statistical significance was claimed for *P*<0.05. Median follow-up is 25.8 months (range: 12–70.2 months).

## RESULTS

**Figure 2:**
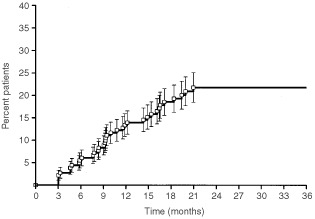
Actuarial incidence (±s.e.) of grade 2–4 late rectal toxicity in the whole group of patients.

Thirty-four (18.7%), one (0.5%) and one (0.5%) patients developed grade 2, 3 or 4 late rectal toxicity, respectively. Median time to onset of late toxicity was 10.1 months (range: 3–22.6 months). The estimated incidence of grade 2–4 late reactions is 21.8±3.2% at 2 years (Figure 2

).

Univariate analysis data are shown in Table 2

**Table 2:**
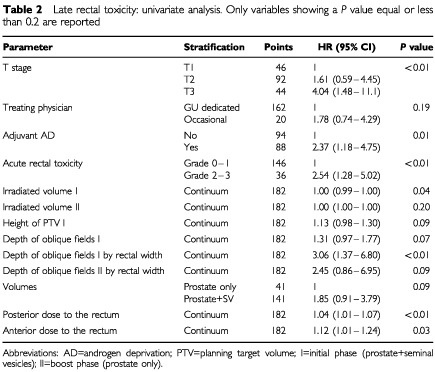
Late rectal toxicity: univariate analysis. Only variables showing a *P* value equal or less than 0.2 are reported

. T stage (*P*<0.01), adjuvant hormonal therapy (*P*=0.01), irradiated volume of the initial phase (*P*=0.04), depth of oblique fields of initial phase by rectal width (*P*<0.01), acute rectal toxicity (*P*<0.01), posterior and anterior doses to the rectum on the central axis (*P*<0.01 and 0.03, respectively) were significant. A trend was found for height of PTV during the initial phase (*P*=0.09), depth of oblique fields of initial phase (*P*=0.07), depth of oblique fields of boost phase by rectal width (*P*=0.09) and prescribed volumes (*P*=0.09).

At multivariate analysis (Table 3

**Table 3:**
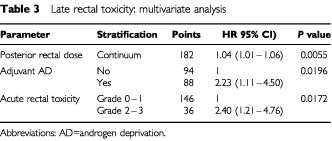
Late rectal toxicity: multivariate analysis

), the posterior dose to the rectum, adjuvant hormonal therapy and acute rectal toxicity were independent predictors of late rectal toxicity. No other variable was significant. In particular anterior dose was not significant as either continuous or categorised variable.

**Figure 3:**
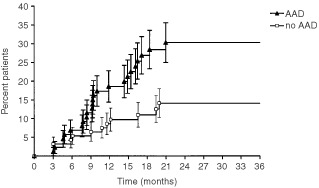
Actuarial incidence (±s.e.) of grade 2–4 late rectal toxicity by adjuvant hormonal treatment.

Patients receiving adjuvant hormones have a risk of grade 2–4 rectal toxicity which is 2.23 times greater (95% CI: 1.11–4.50, *P*=0.0196) than that of patients not receiving hormones. The 2-year estimate of grade 2–4 late rectal toxicity for patients receiving or not adjuvant hormonal treatment were 30.3±5.2% and 14.1±3.8%, respectively (Figure 3

).

## DISCUSSION

Radiation induced late rectal toxicity has been taken as surrogate for dose escalation feasibility of 3DCRT for prostate carcinoma. Contrary to toxicity to the bladder, that is the other organ limiting dose escalation, rectal toxicity usually develops sooner (Mameghan *et al*, 1990) and it has also been shown to be correlated to physical parameters of treatment such as dose and volume (Fiorino *et al*, 2001; Boersma *et al*, 1998). There are, however, important drawbacks. Severe (grade 3 or more) late complications (Table 1) develop in a minority (<1–2%) of patients (Dearnaley *et al*, 1999; Storey *et al*, 2000; Skwarchuk *et al*, 2000; Schultheiss *et al*, 1995). Their rarity makes late toxicity analysis not comparable within small, prospective, single-institution trials. On the other hand, a reliable estimate of grade 2 late rectal toxicity is hampered by several pitfalls. While severe or higher late complications are unlikely to escape documentation and are easily recognised according to the RTOG scale (Table 1), the scoring of less severe complications might be subjective, thus questioning its reproducibility within multicenter trials. This fact also justifies the introduction of modifications in the RTOG scale (Hanlon *et al*, 1997).

The knowledge of individual radiotherapy treatment data is also crucial to analysis. Neoadjuvant hormonal therapy is known to induce modifications of prostate gland volume (Zelefsky and Harrison, 1997; Forman *et al*, 1995; Marcenaro *et al*, 2001). Patients undergoing 3-month neoadjuvant AD may have a reduced risk of late toxicity throughout a more favourable geometry of the treatment of a shrunk prostate gland (Zelefsky and Harrison, 1997). On the other hand, if such shrinkage is not taken into account at radiotherapy treatment planning, an increased risk of complications is also possible (Marcenaro *et al*, 2001; Schultheiss *et al*, 1995).

All these biases may have affected previous reports on late rectal toxicity to some extent (Bolla *et al*, 1997; Sanguineti *et al*, 2000; Lawton *et al*, 2001). Contrary to our previous analysis, we tried to minimise the impact of such biases by considering a homogeneously treated group of patients for whom treatment details were available. Moreover, late reactions were prospectively recorded at a single institution by just two observers (G Sanguineti and P Franzone). Similarly to other authors (Haie-Meder *et al*, 1994) we scored also moderate, grade 2, reactions.

Our results show that rectal tolerance is reduced in presence of adjuvant hormonal therapy. The same conclusion comes also from the study of Fiorino *et al* (2001), where, similarly to our experience, individual dose/volume histograms (DVH) of the rectum were kept in the analysis. In the experience of Fiorino *et al* (2001), AAD along with ICRU total dose and DVHs of the rectum were independent predictors of grade 2+ late rectal bleeding. In particular, patients undergoing AAD had a 2.8 (95% CI: 1.0–7.9) increased risk of grade 2+ late rectal bleeding, which is close to our estimate (Table 3).

The underlying mechanism of such phenomenon is not known. After radiotherapy, chronic pathologic changes occurring in the rectum include fibrosis and vascular insufficiency (Coia *et al*, 1995). Main changes involve the submucosa where atypical fibroblasts, collagen proliferation, thickening of walls of small arteries and telangiectatic vessels can be found. The fact that neoadjuvant AD has little impact on late rectal toxicity compared to adjuvant AD suggests that androgen deprivation may hamper the reparative process of the rectal tissue that is damaged by radiotherapy. Further studies are needed to elucidate this and other aspects such as the duration of AAD and the type of hormonal therapy.

The clinical impact of our findings might be somewhat limited since most of our toxicities were moderate ones (grade 2) althought even less intense late rectal reactions can bother patients quality of life and quality of function (Lilleby *et al*, 1999).

Moreover, since even with conformal radiotherapy, the prescribed total dose to the target is close to the tolerance of neighbouring organs such as rectum and bladder, our findings should be carefully taken into account when combining high-dose 3DCRT and AAD.
